# Intranasal vaccination with extracellular serine proteases of *Leishmania amazonensis* confers protective immunity to BALB/c mice against infection

**DOI:** 10.1186/1756-3305-7-448

**Published:** 2014-09-19

**Authors:** Herbert Leonel de Matos Guedes, Beatriz Lilian da Silva Costa, Suzana Passos Chaves, Daniel Cláudio de Oliveira Gomes, Joshua Daniel Nosanchuk, Salvatore Giovanni De Simone, Bartira Rossi-Bergmann

**Affiliations:** Laboratório de Imunofarmacologia, Instituto de Biofísica Carlos Chagas Filho, Universidade Federal do Rio de Janeiro, 21941-902 Rio de Janeiro, RJ Brazil; FIOCRUZ-Oswaldo Cruz Foundation. Center for Technological Development in Health (CDTS)/National Institute of Science and Technology of Innovation on Diseases of Neglected Populations, 21045-900 Rio de Janeiro, RJ Brazil; Laboratório de Imunologia Celular e Molecular, Núcleo de Doenças Infecciosas, Universidade Federal do Espírito Santo, 29040-091 Vitória, ES Brazil; Departments of Medicine and Microbiology and Immunology, Albert Einstein College of Medicine, Yeshiva University, 10461 Bronx, NY USA; Núcleo Multidisciplinar de Pesquisa UFRJ – Xerém em Biologia (NUMPEX-BIO), Polo Avançado de Xerém – Universidade Federal do Rio de Janeiro, 25245-390 Duque de Caxias, RJ Brazil

**Keywords:** *Leishmania amazonensis*, Murine cutaneous leishmaniasis, Mucosal vaccine, Intranasal route, Extracellular serine proteases

## Abstract

**Background:**

Previously, we demonstrated that unlike subcutaneous or intramuscular vaccination, intranasal vaccination of BALB/c mice with whole *Leishmania amazonensis* antigens leads to protection against cutaneous leishmaniasis. Here, the role of parasite serine proteases in the protective immunity was investigated.

**Findings:**

Serine Proteases were partially purified from both soluble (LaSP-Sol) and extracellular (LaSP-Ex) *Leishmania amazonensis* promastigote extracts by aprotinin-agarose chromatography. BALB/c mice were intranasally immunized with LaSP-Sol and LaSP-Ex prior to infection with *L. amazonensis*. LaSP-Ex but not LaSP-Sol vaccination led to significantly smaller lesions and parasite burdens as compared with non-vaccinated controls. Protection was accompanied by systemic Th1 polarization with increased IFN-γ and decreased IL-4 and IL-10 splenic production. Likewise, increased production of IFN-γ, IL-12 and IL-4 concomitant with decreased TGF-β and TNF-α was locally observed in the infected footpad.

**Conclusion:**

This study indicates that extracellular serine proteases of *L. amazonensis* are strong candidates for a more defined intranasal vaccine against cutaneous leishmaniasis.

**Electronic supplementary material:**

The online version of this article (doi:10.1186/1756-3305-7-448) contains supplementary material, which is available to authorized users.

## Background

Leishmaniasis is a complex of diseases caused by different species of *Leishmania* protozoans affecting over 11 million people worldwide, with an estimated 1.3 million new cases a year. Clinical manifestations vary from a localized cutaneous lesion to lethal visceral infection [1]. Although protective immunity can be acquired following natural infection, three vaccines are currently on the market against canine visceral leishmaniasis [2], and a multisubunit recombinant *Leishmania* vaccine formulated with the MPL-SE adjuvant have progressed to phase-II clinical trials against cutaneous and visceral leishmaniasis [3, 4], no vaccine has been considered safe and/or effective enough for human approval [5]. One of the most extensively studied human vaccines is Leishvacin®, comprised of whole-killed promastigotes of *L. amazonensis*, which have been shown to convert the Montenegro skin test and induce IFN-γ responses in human volunteers, but its efficacy was not confirmed after a controlled phase III clinical trial in Colombia [6], in line with studies in mice and in monkeys showing that adjuvant-free *L. amazonensis* antigens is counter-protective [7, 8].

In common, all the vaccines above were given by subcutaneous or intramuscular (i.m.) routes. Mucosal vaccination is an effective way to induce active immunity against infective agents entering the body through the mucosa, and also an interesting strategy to induce local and peripheral tolerance to otherwise disease-promoting antigens such as allergens in a non-invasive fashion [9]. In this context, the nasal mucosa may work in a way similar to the oral route, but ease of administration, lower antigen dose and lower degradation of the antigen are the main positive factors of its use in relation to the oral route [10].

We previously showed that contrary to the i.m. route, both oral and intranasal (i.n.) vaccination of mice with whole *L. amazonensis* promastigote antigens (LaAg) led to protection against cutaneous leishmaniasis [7, 10, 11]. Since LaAg is a complex antigen, we sought to identify active components for a more defined vaccine. Various serine proteases (SPs) have been demonstrated in *Leishmania* [12–14]. The observation that neutralization of SPs and to a lesser extent cysteine proteases with specific inhibitors rendered LaAg protective via i.m. vaccination, and that i.m. vaccination with partially purified soluble serine proteases enhanced mouse susceptibility to *L. amazonensis* infection [15], we proposed here to evaluate the protectiveness of SPs from intracellular and extracellular parasite extracts against *L. amazonensis* infection using the i.n. route of administration.

## Findings

### Methods

#### Animals

BALB/c mice were originally obtained from Jackson Laboratory (Bar Harbor, Maine), and bred and maintained at our facilities at Universidade Federal do Rio de Janeiro using sterilized bedding, filtered water, and commercial pelleted food, mice were maintained under a controlled temperature. Female mice were used at 6–8 weeks of age in all experiments. Experiments were approved under the protocol # CAUAP180 by the Comitte for Ethical Use of Experimental Animals at CCS/Federal University of Rio de Janeiro (Brazil).

### Parasites

For antigen preparation, *Leishmania amazonensis* (IFLA/BR/67/PH8) routinely isolated from mouse lesions were transformed and maintained as promastigotes at 26°C in M199 medium containing 10% heat-inactivated fetal bovine serum (HIFCS, GIBCO Laboratories, Grand Island, NY). For infection, *L. amazonensis* (MHOM/BR/75/Josefa) transfected with green fluorescent protein (*L. amazonensis*-GFP) were routinely isolated from mouse lesions and maintained as promastigotes at 26°C in M199 medium containing 10% heat-inactivated fetal bovine serum (HIFCS). Parasites were periodically selected for bright fluorescence using 1 mg/mL of geneticin (Sigma) [16].

### LaAg

*L. amazonensis* promastigote antigens (LaAg) were prepared as previously described [15]. Briefly, stationary-growth phase promastigotes were washed three times in phosphate buffered saline (PBS) and subjected to three cycles of freezing and thawing. LaAg was lyophilized, stored at −20°C and reconstituted with PBS immediately prior to use.

### Serine protease purification

The soluble serine protease fraction (LaSP-Sol) and extracellular serine protease fraction (LaSP-Ex) were purified as previously described [12–14]. The soluble serine protease fraction (LaSP-Sol) was purified from soluble extract of promastigotes [12] and the extracellular serine protease fraction (LaSP-Ex) was purified from parasite culture supernatants [13]. For that, 4 × 10^11^ promastigotes (2 ×10^7^/mL) were cultured in 2 L of Brain Heart Infusion (BHI) medium containing 10% HIFCS to the stationary-phase of growth. Promastigotes were washed three times in phosphate buffered saline (PBS) and subjected to three cycles of freezing and thawing in Tris–HCl buffer, pH 7.5, containing 5 mM CaCl_2_ and the cell lysate was centrifugated (100,000 × *g* for 30 min at 4°C) to remove the non-soluble aggregates, thus producing a soluble fraction. The culture supernatants (2 L) were precipitated overnight with 45% (NH_4_)_2_SO_4_ and centrifuged (10,000 × *g* for 30 min at 25°C). The pellet was suspended in 100 mL of 10 mM Tris–HCl buffer, pH 7.5, containing 5 mM CaCl_2_, dialyzed four times against 2 L of the same buffer, and centrifuged (20,000 × *g* for 30 min at 4°C) producing extracellular fraction.

The soluble fraction or extracellular fraction were loaded onto a pre-equilibrated (10 mM Tris–HCl buffer, pH 7.5, containing 5 mM CaCl2) aprotinin-agarose affinity column (2.5 ml; Sigma-Aldrich). After washing with 20 bed volumes, the material was eluted with 10 mM Tris–HCl buffer, pH 7.5, containing 1.5 M NaCl. One mL fractions were collected on ice and the effluents absorption at 280 nm was monitored to detect protein peak. Protein fractions were then pooled (LaSP-Sol or LaSP-Ex), dialyzed against phosphate buffered saline (PBS), and the protein concentration was quantified by the Lowry method.

### Immunization, infection challenge and evaluation of disease progression

Animals received 20 μg of LaSP-Sol or LaSP-EX n 20 μL of PBS by instillation of 10 μL in each nostril using a micropipette adapted with a polystyrene microtip. A booster dose was given 7 days later [10]. Controls received PBS alone. Seven days after the vaccine boost, animals were subcutaneously injected in the hind footpad with 2 × 10^5^ stationary-phase *L. amazonensis*-GFP promastigotes (7 days of culture). Alternatively, for a lower virulence infection that allowed non-vaccinated controls not to ulcerate (approximately 3 mm of tickeness) before 100 days of infection, animals were infected with 2 × 10^5^ promastigotes at the beginning of stationary-phase of growth (4 days of culture). Lesion sizes were periodically measured with a dial caliper and expressed as the difference between the thicknesses of infected and contralateral non-infected footpads. For parasite load determination, the fluorescence intensity of the tissue homogenates were determined as described previously [11]. Briefly, at the end of the experiments each infected foot was skinned and individually homogenized in 1 mL of PBS using a tissue grinder. Tissue debris was removed by gravity sedimentation for 5 min, and then 2-fold serially diluted in PBS in triplicates for fluorescence intensity measurements using a plate-reader fluorimeter (FLX 8000, Bio-Tek) at 435 nm excitation and 538 nm emission.

### Cytokines

For splenic cells, spleens were isolated at 7 days after infection, and single-cell suspensions were adjusted to 4 × 10^6^ per ml in Dulbecco’s minimal essential medium containing 10% HIFCS, 20 mM HEPES, 50 μM 2-mercaptoethanol, 2 mM L-glutamine, 50 U/ml penicillin, 50 μg/ml streptomycin sulfate (Sigma-Aldrich) and 1 mL samples were plated in 24-well tissue culture. Cells were cultured for 48 h in the presence of 5 μg/mL of LaAg, when they were centrifuged and the supernatants collected. For lymph node cells, at 7 days after infection the popliteal lymph nodes were isolated and the cells were cultured as above using 50 ug/mL of LaSP-Sol or LaSP-Ex. For *in situ* production, infected footpads were isolated, skinned, weighed, teased and individually homogenized in 1 mL of PBS using a glass tissue homogenizer. The footpad homogenates were centrifuged (10 min, 20 000 × g at 4°C) and the supernatants collected. For cytokine quantification, supernatants prepared as above were assayed for TGF-β, IFN-γ, IL-10, IL-4, IL-12 and TNF-α by ELISA following the manufacturer’s instructions (R&D Systems, Minneapolis, USA). For TGF-β, the supernatants were pre-heated to 80°C for 5 min prior to the assay [7].

### Statistical analysis

Each experiment was performed three times and similar patterns of results were achieved. Due to expected variations among lesion growth rates in PBS controls, the result is representative of one experiment. The differences between vaccinated and non-vaccinated groups were statistically determined by Student *t*-test or Anova using the GraphPad InStat software, and were considered significant when p ≤ 0.05.

## Results

### Intranasal vaccination with LaSP-Ex but not LaSP-Sol protects mice against infection

**Figure 1:**
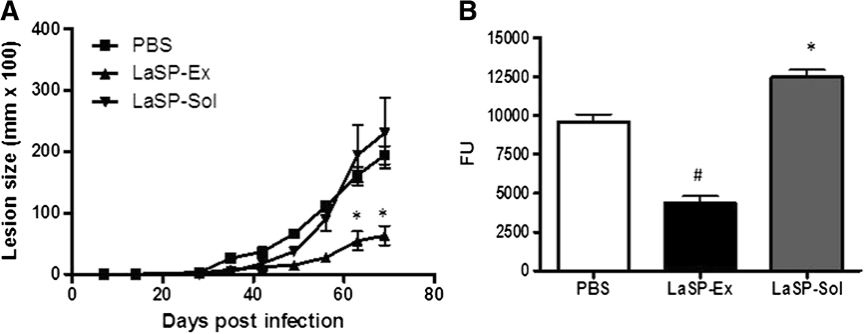
**Efficacy of i.n. LaSP-Sol and LaSP-Ex against** ***L. amazonensis*** **infection.** BALB/c mice received 20 μg of LaSP-Sol or LaSP-Ex by the i.n. route on days −14 and −7 of infection. Non-vaccinated controls received PBS alone. On day 0, animals were infected with 2 × 10^5^ stationary-phase promastigotes of *L. amazonensis*-GFP (7 days of culture). **A)** Lesion sizes were measured at the indicated days and are expressed as the difference of thickness between non-infected and infected footpads. **B)** The parasite loads were measured on day 70 of infection and are expressed as fluorescence units (FU). The data displayed are representative of three independent experiments producing the same result profile. Means ± SD (n = 5). *p ≤ 0.05 and ^#^p ≤ 0.01 in comparison to PBS controls.

**Figure 2:**
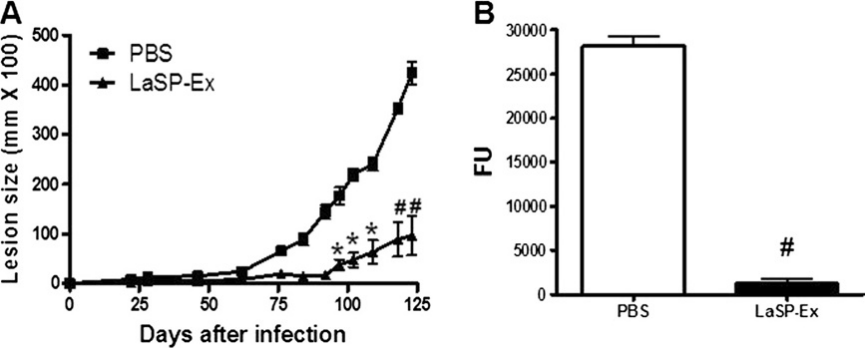
**Protection duration of i.n. LaSP-Ex upon a less virulent infection.** BALB/c mice were vaccinated with LaSP-Ex and seven days after the booster animals were challenged with 2 × 10^5^ at the beginning of stationary-phase *L. amazonensis*-GFP (4 days of culture) and monitored as described for Figure 1. **A)** Lesion sizes. **B)** Parasite loads were measured on day 125 of infection. Means ± SD (n = 5). *p ≤ 0.05 and ^#^p ≤ 0.01 relative to PBS controls. The data displayed is representative of two independent experiments.

The efficacy of i.n. vaccination with LaSP-Ex and LaSP-Sol were evaluated in *L. amazonensis*-challenged mice seven days after a booster. Vaccination with LaSP-Sol did not significantly alter the course of infection as compared with controls (Figure 1A). On day 70 of infection, when there was a slight lesion outgrowth, the parasite loads were higher than controls (Figure 1B), indicating that i.n. LaSP-Sol led to increased susceptibility to infection. Opposite to LaSP-Sol, LaSP-Ex effectively controlled lesion growth (Figure 1A) and parasite Load (Figure 1B). In the low virulent infection, we followed infections until day 125. We observed a high level of protection by delay of growth curve and control of the lesion size (Figure 2A). The parasite burden in the footpads was significantly lower than controls (Figure 2B), confirming the lesion control finding.

### Intranasal LaSP-Ex induces Th1 systemic polarization

**Figure 3:**
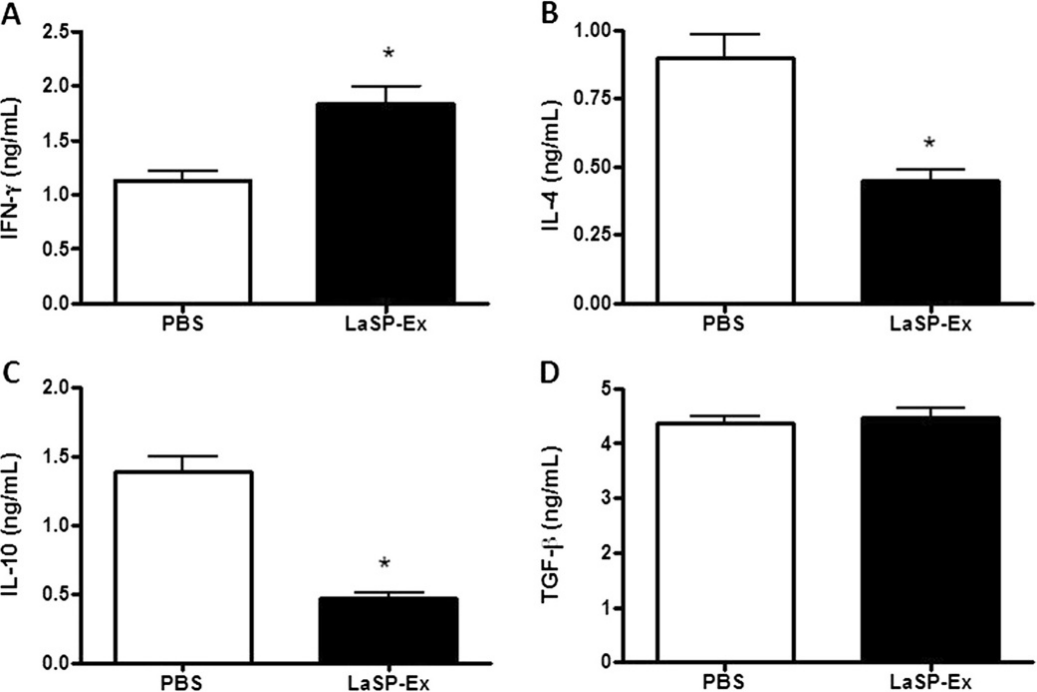
**LaSP-EX induces Th1 systemic polarization.** Mice were vaccinated and infected as described for Figure 2. At day 7 of infection, their spleen cells were re-stimulated *in vitro* with LaAg (5 μg/mL), and after 48 hours of culture the levels of IFN-γ **(A)**, IL-4 **(B)**, IL-10 **(C)** and TGF-β **(D)** were measured in the supernatants. The data displayed is representative of three independent experiments. Means ± SD (n = 5). *p ≤ 0.05 in relation to PBS.

We evaluated the cytokine production by spleen cells of LaSP-Ex-vaccinated animals at an early stage of infection (7 days after challenge). Upon antigen (LaAg)-stimulation, cells form vaccinated mice produced increased IFN-γ (Figure 3A) and decreased levels of IL-4 and IL-10 (Figure 3B and C), whereas TGF-β was not modulated (Figure 3D). The cytokine levels from LaSP-Sol-vaccinated mice were similar to non-vaccinated PBS controls (not shown), suggesting that the failure of this group may be related to lack of a proper systemic cytokine modulation. At the same time, these findings suggest that protective immunity of LaSP-Ex was associated with a Th1-skewed cytokine production.

### Intranasal LaSP-Ex protection is accompanied by mixed *in situ*cytokine responses

**Figure 4:**
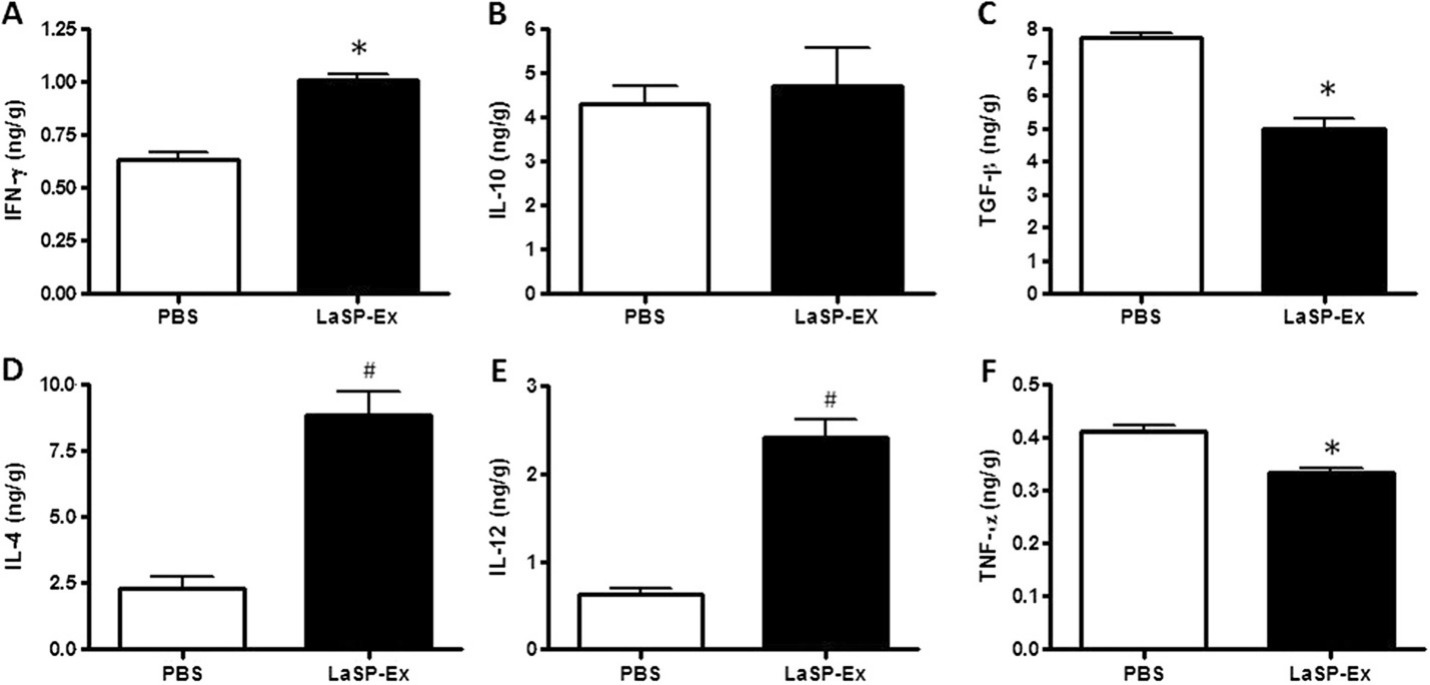
**In situ cytokine profile.** Mice were i.n. vaccinated with LaSP-Ex and infected with *L. amazonensis*-GFP as described for Figure 2. On day 125 of infection, the levels of IFN-γ **(A)**, IL-10 **(B)**, TGF-β **(C)**, IL-4 **(D)**, IL-12 **(E)** and TNF-α **(F)** were measured in the lesion homogenates, and expressed as ng per g of footpad. The data displayed is representative of three independent experiments. Means ± SD (n = 5). *p ≤ 0.05 and ^#^p ≤ 0.01 in comparison to PBS controls.

To investigate the mechanism of protection of i.n. LaSP-Ex, the *ex vivo* production of key pro-inflammatory (IFN-γ, IL-12 and TNF-α) and anti-inflammatory (IL-4, IL-10 and TGF-β) cytokines were evaluated in the footpads at the end of the experiments. Figure 4 shows a higher production of IFN-γ, IL-4 and IL-12 (Figure 4A, D and E) concomitant with reduced TGF-β and TNF-α (Figure 4C and F) and unaltered IL-10 (Figure 4B) in relation to non-vaccinated controls, indicating a mixed pro- and anti-inflammatory local cytokine response.

## Discussion

Few vaccines that have been administered by the mucosal route are able to stimulate effective cell-mediated immune responses, the exceptions being invasive bacterial vaccines [17]. Defined leishmanial antigens such as recombinant LACK [10, 18] and Leish-111f [19] were evaluated as vaccines by intranasal route against *Leishmania* infection. Recombinant LACK [18] and Leish-111f [19] only induced protection by intranasal route when associated with cholera toxin as adjuvant. However, cholera toxin is not approved for human use as a mucosal adjuvant [20].

In this work, we evaluated the protectiveness of intranasal vaccination using two new leishmanial serine protease fractions, LaSP-Sol and LaSP-Ex, without any adjuvant against *L. amazonensis* infection. In previous studies, we showed that those two protease fractions display different protein contents [12, 13]. Subsequently we showed that intramuscular vaccination with LaSP-Sol leads to enhanced susceptibility to infection, in a manner associated with its capacity to stimulate IL-10 and TGF-beta production by immune cells from 7-day infected mice [15]. In the present work, using a similar *in vitro model,* we observed that contrary to LaSP-Sol, LaSP-Ex does not stimulate the production of TGF-β, and actually inhibits the spontaneous production of IL-10 (Additional file 1: Figure S1). The observed differential antigenicity between these two SP fractions prompted their comparison as mucosal vaccines. Interestingly, LaSP-Sol is comparable to LaAg by i.m. immunization [7, 15], but not for i.n. immunization (Figure 1). On the other hand, i.n. LaSP-Ex vaccination promoted resistance to *L. amazonensis* challenge infection (Figures 1 and 2), similar to i.n. LaAg vaccination [10]. The ability of adjuvant-free LaSP-Ex to induce protection is very encouraging, as this is a more defined antigen fraction.

Immunity against *Leishmania* parasites depends largely on activation of cell-mediated responses, and gamma interferon and dendritic cell-derived IL-12 has been shown to play a crucial role in this process [21]. Whereas in *L. major* murine infection, IFN-γ production is more critical at the beginning of infection [22], in *L. amazonensis* infection IFN-γ requirement seems particularly important in the later stages [23]. Here, we have shown the increased production of IFN-γ (Figure 3A) and decreased production of IL-4 and IL-10 (Figure 3B and C) in spleen cells after seven days of infection that indicates the systemic Th1 polarization induced by LaSP-Ex intranasal vaccination. LaSP-Ex also led to increased peripheral IFN-γ (Figure 4A) in the site of infection as compared to non-vaccinated controls, and this is probably the result of increased IL-12 (Figure 4E), which is required not only to initiate but also to maintain Th1 cell responses and resistance to *L. major* [24].

Conversely, macrophage deactivating cytokine TGF-β has been associated with disease [7, 25, 26]. Also, i.n. LaSP-Ex vaccination led to a decreased TGF-β production (Figure 4C) in the infected footpads. This is in line with our previous studies showing the reversal of TGF-β production and decreased susceptibility of BALB/c mice given i.m. LaAg devoid of SP activity [15]. The decrease of TNF-α (Figure 4F) could be related to the smaller lesion observed in the vaccinated group, as it has been demonstrated in humans that larger lesions are associated with increase of TNF-α [27]. IL-4 is required for an optimal Th1 response against *L. major*, because of the increase in the production of IL-12 by dendritic cells [28]. The increase of IL-4 (Figure 4D) in the footpad of the vaccinated group suggests the participation of this cytokine in the lesion resolution. Overall, the cytokine balance indicates that even at this late stage of infection (day 125) it tends to be a pro-inflammatory local response.

LaSP-Ex is an exogenous purified antigen from *L. amazonensis* culture. Exogenous antigens from *Leishmania major* culture (SEAgs) have been evaluated as vaccine candidates against *L. major, L. donovani and L. braziliensis* infections in BALB/c mice [29, 30]. The immunization with adjuvant-free SEAgs by parenteral route induced protection against *L. major* and *L. donovani*, but not against *L. braziliensis*. Two components of SEAgs, GP63 and nucleoside hydrolase, induced protection against *L. major* infection [29–31]. The identity of serine proteases in LASP-Ex remains unknown. We cloned oligopeptidase B (OPB) [32] and oligopeptidase B2 (OPB2) [33] from *Leishmania amazonensis* and we predicted the molecular weight for OPB and OPB2 as 83.49 KDa and 103.92 KDa, respectively. We found two protein bands with similar molecular mass in the extracellular serine proteases fraction of *L. braziliensis* [14], suggesting the presence of OPB and OPB2 in the extracellular serine proteases fraction. Oligopeptidase B is an important virulence factor for trypanosomatids [34, 35]. Studies evaluating the components of LaSP-Ex and immunogenicity of recombinant OPB and OPB2 are under way and may help clarify their participation in the protective effect of LaSP-Ex. The evaluation of proteases as antigens for immunization is very important to development of new vaccines against infectious diseases [34, 36].

## Conclusion

We have previously demonstrated the feasibility of both oral and intranasal (i.n.) vaccination with particulated leishmanial antigens (LaAg) in protecting BALB/c mice against *L. amazonensis* [10, 11] and intranasal DNA encoding LACK vaccination protecting BALB/c mice against *L. amazonensis* [11]. In this manuscript, we demonstrate that a more defined antigen, extracellular serine proteases of *L. amazonensis*, is protective via an intranasal route encouraging further work on this second-generation vaccine.

## Electronic supplementary material

Additional file 1: Figure S1: In vitro cytokine production by immune cells stimulated with LaSP-Sol and LaSP-Ex. Lesion-draining lymph node cells were isolated from 7-day infected mice and re-stimulated *in vitro* with 50 μg/mL of soluble serine proteases of LaAg (LaSP-Sol) or extracellular serine proteases fraction (LaSP-Ex). After 48 h, the levels of IL-10 (A), TGF-β (B) and IFN-γ (C) were measured in the cell supernatants. Means ± SD (n = 5). *p ≤ 0.05 and ^#^p ≤ 0.01 in relation to unstimulated controls (medium). (PPTX 54 KB)

## Abbreviations

i.n.: Intransal
i.m: Intramuscular
LaSP-Sol: Soluble Serine proteases from *Leishmania amazonensis* promastigotes
LaSP-Ex: Extracellular serine proteases from *Leishmania amazonensis* promastigotes supernantant of culture
SP: Serine proteases.
