# Supplementary figures and images for: Intranasal vaccination with extracellular serine proteases of Leishmania amazonensis confers protective immunity to BALB/c mice against infection
Source: Parasit Vectors. 2014 Sep 19;7:448. doi: 10.1186/1756-3305-7-448 (PMC4261548; doi:10.1186/1756-3305-7-448)

## Slide 1
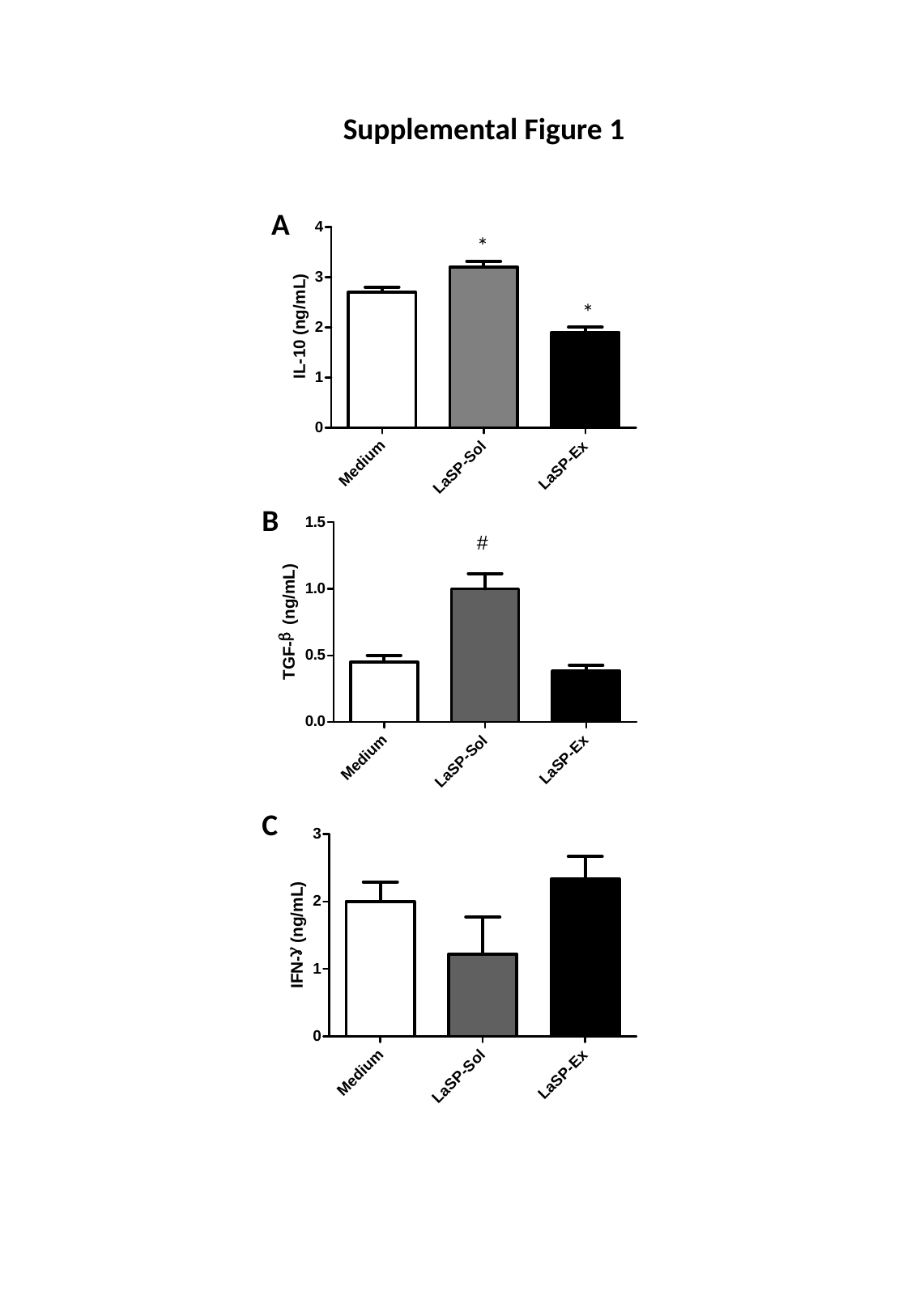

Supplemental Figure 1
A
*
*
**
B
#
C

Supplement: Supplementary file 1 — Additional file 1: Figure S1: In vitro cytokine production by immune cells stimulated with LaSP-Sol and LaSP-Ex. Lesion-draining lymph node cells were isolated from 7-day infected mice and re-stimulated in vitro with 50 μg/mL of soluble serine proteases of LaAg (LaSP-Sol) or extracellular serine proteases fraction (LaSP-Ex). After 48 h, the levels of IL-10 (A), TGF-β (B) and IFN-γ (C) were measured in the cell supernatants. Means ± SD (n = 5). *p ≤ 0.05 and #p ≤ 0.01 in relation to unstimulated controls (medium). (PPTX 54 KB) [file 13071_2014_1619_MOESM1_ESM.pptx]
